# Arachnoid cyst in a patient with psychosis: Case report

**DOI:** 10.1186/1744-859X-6-16

**Published:** 2007-06-28

**Authors:** Joaquim Alves da Silva, Alexandra Alves, Miguel Talina, Susana Carreiro, João Guimarães, Miguel Xavier

**Affiliations:** 1 Depart. Psychiatry and Mental Health, Faculty Medical Sciences – UNL Calçada da Tapada, 155, 1300-Lisbon, Portugal; 2 Depart. Psychiatry – Hospital S. Francisco Xavier, 1400-Lisbon, Portugal; 3 Depart. Neurology, Faculty Medical Sciences – Hospital Egas Moniz, 1400-Lisbon, Portugal

## Abstract

**Background:**

The aetiology of a psychotic disturbance can be due to a functional or organic condition. Organic aetiologies are diverse and encompass organ failures, infections, nutritional deficiencies and space-occupying lesions. Arachnoid cysts are rare, benign space-occupying lesions formed by an arachnoid membrane containing cerebrospinal fluid (CSF). In most cases they are diagnosed by accident. Until recently, the coexistence of arachnoid cysts with psychiatric disturbances had not been closely covered in the literature. However, the appearance of some references that focus on a possible link between arachnoid cysts and psychotic symptoms has increased the interest in this subject and raised questions about the etiopathogeny and the therapeutic approach involved.

**Clinical presentation:**

We present the clinical report of a 21-year-old man, characterised by the insidious development of psychotic symptoms of varying intensity, delusional ideas with hypochondriac content, complex auditory/verbal hallucinations in the second and third persons, and aggressive behaviour. The neuroimaging studies revealed a voluminous arachnoid cyst at the level of the left sylvian fissure, with a marked mass effect on the left temporal and frontal lobes and the left lateral ventricle, as well as evidence of hypoplasia of the left temporal lobe. Despite the symptoms and the size of the cyst, the neurosurgical department opted against surgical intervention. The patient began antipsychotic therapy and was discharged having shown improvement (behavioural component), but without a complete remission of the psychotic symptoms.

**Conclusion:**

It is difficult to be absolutely certain whether the lesion had influence on the patient's psychiatric symptoms or not.

However, given the anatomical and neuropsychological changes, one cannot exclude the possibility that the lesion played a significant role in this psychiatric presentation. This raises substantial problems when it comes to choosing a therapeutic strategy.

## Background

Psychotic disorders which may be caused by either functional or organic conditions, are clinical entities characterised by changes in perception and thinking, thus interfering with the patient's social performance [1].

In DSM-IV, psychosis with an organic aetiology is named "Psychotic Disorder due to a General Medical Condition" and has two subgroups: i) with hallucinations, and ii) with delusions [2]. Traumatisms or structural changes of the brain such as space-occupying lesions; biochemical changes (including intoxication with drugs); organ failure; infections; and nutritional deficiencies are all examples of causes of psychoses that are secondary to a general medical condition [1,3-6].

Arachnoid cysts are benign space-occupying lesions containing CSF. They are rare lesions and account for only 1% of all intracranial space-occupying lesions [7]. From an etiological point of view we should distinguish between true cysts (of a congenital nature) and false ones, which are secondary to the post-inflammatory accumulation of CSF during cranial traumatisms, infections or intracranial haemorrhages [7,8]. Arachnoid cysts can appear in any area of the central nervous system, though they are more frequent in the Sylvian fissure, where they are found in about 50% of cases [8]. They occur roughly twice as often on the left side as they do on the right, although the reason for this is unknown [7-9] and there is a preponderant ratio of 3:1 in male as opposed to female patients [9].

Arachnoid cysts are often diagnosed before adulthood (60–90% prior to the age of 16) [8]. In most cases diagnosis is accidental, and it may even result from a fortuitous discovery during a post-mortem examination [7,8,10].

Three mechanisms for their expansion have been described: i) unidirectional valvular mechanism; ii) displacement of liquid due to an increased osmotic gradient within the cyst; and iii) secretion of liquid by the cells that compose the walls of the cyst [7,8]. The clinical picture of these anomalies varies depending on their location and the patient's age. During the paediatric period hydrocephaly or cranial deformation are the most frequent manifestations, whereas in adults, headaches and convulsive episodes are the most common [8]. Other signs and symptoms include ataxia, ocular alterations, focal signs, dizziness, and altered memory [8,11].

Although arachnoid cysts are classically considered to be incidental lesions when found in people with psychiatric disorders (and no elementary neurological signs) [11], some articles point to the existence of a putative causal relationship [11-21].

The discovery of an arachnoid cyst in a person with a psychotic disorder raises diagnostic and therapeutic problems that are extremely significant from a clinical point of view [11,12,15,18-20].

## Case Presentation

A 21-year-old man went to the emergency department of São Francisco Xavier Hospital (Lisbon) saying that he had appendicitis and needed an operation. He also said that his appendix and his liver were interfering with his voice. According to his mother, for the last three years the patient had displayed periods of behavioural changes, with aggressive behaviour and unwarranted laughter. Recently, he had been fired from several jobs for being late. The patient justified his behaviour by saying that he couldn't sleep at night, and described what seemed to be complex auditory hallucinations in the second and third persons with a depreciatory content.

In the previous two months the clinical picture had deteriorated, with disorganised thoughts and "periods in which he wasn't there", during which he did not answer any questions or initiate any conversation. According to the patient himself, at such times, he was perplexed because the words people said appeared to make no sense.

During the mental state examination, the patient was alert and oriented in space and time. He displayed delusions with a hypochondriac theme that focused on concerns about the state of his liver and his appendix, and auditory/verbal hallucinations with a depreciatory content. The patient was euthymic, and his feelings were appropriate, with no blunting or flattening. He did not display any insight into his condition. The neurological exam did not reveal any changes and the Mini-Mental State Examination [22] was normal (29/30).

His prior medical history included a head trauma at the age of 16 that had been caused by a motorcycle accident and had apparently not been serious. No cranial computer tomography (CT) had been done at that time. The patient admitted to a regular consumption of cannabis since the age of 13, together with alcohol abuse that had recently worsened. He also had a sporadic consumption of cocaine and methylenedioxymethamphetamine (MDMA).

His family history included a suicide attempt by his half-brother a few months before, which had not been associated with any psychotic condition.

The blood tests were normal except for the toxicological traces, which revealed the presence of cannabinoids in the urine sample (52 ng/ml).

The patient was compulsorily admitted to the hospital under the terms of the Portuguese Mental Health Law.

Despite the fact that the cannabinoid levels became normal during the first few days of his stay in the hospital, the patient's psychotic symptoms persisted.

**Figure 1:**
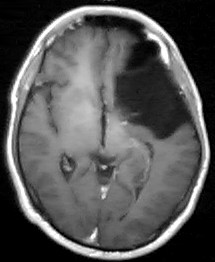
Cranial MNR image in a horizontal plane, showing a voluminous arachnoid cyst in the left sylvian fissure. It also shows the enlarged frontal sinus in contact with the anterior region of the cyst.

**Figure 2:**
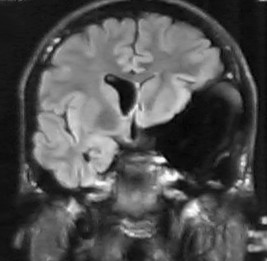
Cranial MNR image in a coronal plane. The mass effect on the left lateral ventricle and on the left temporal lobe (with hypoplasia) determined by the arachnoid cyst is visible.

**Figure 3:**
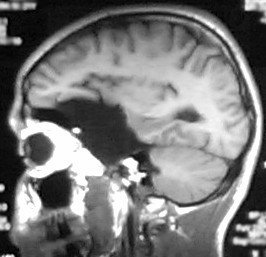
Cranial MNR image in a sagittal plane, showing the orbitofrontal portion of the cyst.

A cranial CT revealed the presence of an arachnoid cyst at the level of the left Sylvian fissure, with a marked mass effect on the left temporal and frontal lobes and the left lateral ventricle. There was also an extensive pneumatisation of the left frontal sinus. A cranial nuclear magnetic resonance (NMR) was performed in order to get a more detailed image. It confirmed the nature of the lesion and revealed the existence of a left temporal lobe hypoplasia that was associated with the arachnoid cyst (Figures 1, 2, 3).

It also showed that the left frontal sinus, which was more developed, was in contact with the arachnoid cyst. An EEG revealed unspecific changes in the median temporo-parietal zones, which were more widespread on the left side. A neuropsychological examination showed various alterations, with impairment of verbal memory, attention, ability to plan and increased impulsiveness with a tendency towards anti-social behaviour.

The patient started antipsychotic therapy with risperidone 2 mg *tid*. Due to the fact that no links between arachnoid cysts and psychotic symptoms have been clearly established and no focal or intracranial hypertension signs were observed, the neurosurgical department concluded that even tough there was a mass effect, the risk of operating was higher than the potential benefits.

The psychotic symptoms improved progressively during the stay, with amelioration of the psychomotor agitation and remission of the auditory/verbal hallucinations. Although it was clear that there was a significant improvement in relation to the delusional hypochondriac ideas, a complete remission of these symptoms was not achieved.

The patient was discharged after a 4-week inpatient stay and received follow-up outpatient care with psychiatric and neurosurgical appointments. Three months after discharge, the patient was working part-time and attending a technical course on computer hardware. He showed the same psychotic symptoms and remained without any insight into his condition.

## Discussion

This patient's clinical picture is characterised by the insidious development of psychotic symptoms: delusional ideas with a hypochondriac content; complex auditory/verbal hallucinations in the second and third persons; and behavioural changes with aggressive behaviour.

The patient did not display the typical characteristics which make it easier to distinguish between non-organic psychosis and organic psychosis such as abnormal vital signs, recent memory changes, age above 40, disorientation and an altered state of consciousness [1,23].

Given the existence of toxic levels of cannabinoids in the patient's urine it is not possible to exclude the hypothesis of a psychosis with a toxic aetiology. However, there was no substantial improvement once the cannabinoids values fell to normal levels.

The anomalous pneumatisation of the frontal sinus found next to the cyst sheds some light into its origin by suggesting a probable congenital malformation instead of a secondary aetiology related to the traumatism which the patient suffered at the age of 16.

In fact, it is difficult to be sure whether we are in the presence of an organic psychotic disorder or of a simple coincidence in which the arachnoid cyst is just an 'innocent bystander' to the development of a functional psychosis.

Although the cyst seems to be congenital, it did not cause any symptoms earlier in life. Nevertheless arachnoid cysts may enlarge and interfere with adjacent neural structures or CSF circulation [7]. The mass effect shown on the neuroimaging studies suggests that this might be the case, and what started as an 'innocent bystander' may not be so innocent after all.

Remission of symptoms following surgical treatment [21], association of psychiatric symptoms with neurological changes [11,12], advanced age, absence of family history, evidence of compression of the temporal lobe and neighbouring structures [12]., and changes in the neuropsychological and neurophysiological tests [11] are all mentioned as factors that suggest an etiologic relationship of arachnoid cysts to the psychiatric disorders. The presence of some of these factors – particularly the evidence of hypoplasia of the left temporal lobe (figure 2), and the neuropsychological changes compatible with those described for orbitofrontal lesions (figure 3) [24] – strengthens the possibility that this lesion played a part in the etiopathogeny of the psychotic symptoms.

Other cases of psychoses that are associated with arachnoid cysts have been described in patients with an injured left temporal lobe [11-13,16,18-20]. Structural changes of the temporal lobe, both at the level of the median structures and of the temporal circumvolution, have been associated with schizophrenia [25].

The patient said there were periods in which the words people said appeared to make no sense. This description is compatible with a dysphasia, which in structural terms could mean that Wernicke's region was compromised by the direct mass effect of the cyst. However, during the inpatient stay the patient did not report any such episodes.

According to current clinical practice, there are two aspects to the therapeutic approach of organic psychotic disorders: i. controlling the symptoms and ii. correcting the etiological situation. In the specific case of arachnoid cysts the need for a surgical approach is neither clear nor uniform: intracranial hypertension or progressive hydrocephaly are usually the only situations where surgery is mandatory [7]. If neither of these indications is present and there are no focal neurological signs, given both the morbidity associated with surgery and the fact that this type of lesion can disappear spontaneously, the attitude is generally conservative [7]. However, an analysis of the literature shows that although this is the most common choice, there have been cases in which surgical intervention was specifically adopted as a therapeutic approach to the psychotic symptoms.

The first patient described in the literature with an acute psychotic disturbance and an arachnoid cyst in the left temporal region, showed a total remission of symptoms after the cyst had been surgically removed [18]. Colameco *et al *[14] describe the case of a patient with episodes of derealization and emotional lability associated with the presence of an arachnoid cyst, in which the symptoms also displayed total remission following the cyst's removal. In a case series by Kohn *et al *[11] that describes eight patients with arachnoid cysts associated with psychiatric disturbances, only one of the cases with psychotic symptoms underwent surgery to remove the cyst. Curiously enough, this patient was the only to experience complete remission of his symptoms. In a recently described case of atypical psychosis associated with an arachnoid cyst, Vakis *et al *[20] found intermittent rises in the intracranial pressure. Although these rises did not result in any neuroimaging changes, the authors considered them to be a plausible etiopathogenic factor in the appearance of the psychotic symptoms in that particular female patient. The surgical removal of the lesion was followed by a clear improvement. Wong *et al *[21] describe the interesting case of a female patient with an arachnoid cyst in the trigone of the right lateral ventricle, which was associated with very short psychotic episodes that arose after the patient had been lying down in bed for 1–2 hours. They called these episodes 'positional psychosis', and suggested that the decubitus position led the cyst to cause a local ischemia in the temporal horn, with the consequent appearance of psychotic symptoms. In this case, it was also decided to operate the lesion, and this led to the total disappearance of the psychotic symptoms in just four days.

In other cases [12,13,15,16] a conservative choice was made and the only treatment was pharmacotherapy with antipsychotics, leading to relative improvements in the psychotic symptoms. However, patients in whom neuropsychological alterations were initially described did not improve much during the follow-up period [13,15].

In the case we present, risperidone was selected due to the good results it has shown in psychosis associated with a general medical condition, including a case of psychosis associated with an arachnoid cyst [13,26].

## Conclusion

It is difficult to tell whether the lesion was innocent or not regarding this patient's overall psychiatric picture. Having said this, the serious changes to the left frontal and temporal lobes with compatible neuropsychological changes, leads to the conclusion that the possibility that the lesion played a part in the etiopathogeny of the psychotic symptoms cannot be excluded.

Given the growing number of cases described in the literature, the psychotic symptoms in patients with this type of lesion cannot be unquestionably seen as a coincidence. Furthermore, in the cases in which it was decided to intervene surgically there was an extremely fast remission of the psychotic symptoms [14,18,20,21].

A more in-depth study of this type of cases is thus required in order to make it possible to optimise the therapeutic approach in cases involving the coexistence of arachnoid cysts and psychosis.

## Competing interests

The author(s) declare that they have no competing interests.

## Authors' contributions

JAS reviewed the existing literature and drafted the manuscript.

AA helped to draft the manuscript.

SC and MT have made substantial contributions to acquisition and interpretation of clinical data.

JG conducted the neurological evaluation and interpreted the clinical data.

MX reviewed the manuscript and contributed to the writing.

All authors read and approved the final manuscript. Moreover, all authors were involved in the care of the patient described in this case report.
